# Guessability of standard pharmaceutical pictograms in members of the Nigerian public

**DOI:** 10.1016/j.rcsop.2023.100240

**Published:** 2023-03-10

**Authors:** Samirah N. Abdu-Aguye, Amina M. Sadiq, Aishatu Shehu, Elijah N.A. Mohammed

**Affiliations:** a Department of Clinical Pharmacy & Pharmacy Practice, Ahmadu Bello University, Zaria, Nigeria; b Department of Pharmacology & Therapeutics, Ahmadu Bello University, Zaria, Nigeria; c Department of Clinical Pharmacy & Pharmacy Practice, Igbinedion University, Okada, Nigeria

**Keywords:** Audiovisual aids, Comprehension, Cross-sectional studies, Nigeria

## Abstract

**Background:**

Pharmaceutical pictograms are standardized images used to visually convey medication instructions. Very little is known about the ability of Africans to interpret these images.

**Objectives:**

Thus, the aim of this study was to assess the guessability (ability to correctly guess meaning) of selected International Pharmaceutical Federation (FIP) and United States Pharmacopoeia (USP) pictograms in members of the Nigerian public.

**Methods:**

A cross-sectional survey was carried out between May and August 2021 on 400 randomly sampled members of Nigerian public. Selected pictograms (24 FIP and 22 USP pictograms) were grouped and printed on A3 sheets of paper which were used to interview members of the public who fulfilled the study's' eligibility criteria. Respondents were asked to guess the meanings of either the FIP or USP pictograms, and their answers written down verbatim. Descriptive and inferential statistics were used to report the data collected.

**Results:**

Four hundred respondents were interviewed, with 200 respondents each assessing the guessability of the FIP and USP pictograms. The guessability of assessed FIP pictograms ranged between 3.5 and 95%, while that for the USP pictograms was 27.5–97%. Eleven FIP and Thirteen USP pictograms respectively achieved the International Organization for Standardization (ISO) comprehensibility cutoff point of 67%. Guessing performance (the total number of pictograms correctly guessed by an individual) of respondents that assessed the FIP pictograms was significantly associated with their age (*p* = 0.044) and highest level of education completed (*p* = 0.003). For the USP pictograms, guessing performance was only significantly associated with the highest educational level completed (*p* < 0.001).

**Conclusions:**

Guessability of both pictogram types varied widely, but the guessability of the USP pictograms was generally better than that for the FIP pictograms. Many of the tested pictograms may however need to be redesigned before they can be correctly interpreted by members of the Nigerian public.

## 1 Introduction

Pharmaceutical pictograms are standardized images targeted at patients or consumers that help to visually convey medication instructions, precautions, and/or warnings. They are one of the more commonly used strategies by pharmacists and other healthcare professionals to convey medication related information to patients especially those with low educational levels or for whom English is a second language. When pictograms are used together with adequate written or verbal medication counselling, they have been shown to improve patient comprehension and recall,^1^, ^2^, ^3^ enhance medication adherence^4^ and reduce pediatric dosing errors.^1^

Various types of pharmaceutical pictograms exist that were developed by various organizations and/researchers. Pictogram libraries have been developed and are currently maintained by the United States Pharmacopoeia (USP) convention, the Risk benefit Assessment of Drug Analysis and Response (RAD-AR) council of Japan and the International Pharmaceutical Federation (FIP).^5^ Several researchers have also adapted and or developed pictograms for specific populations and/ disease states. The USP pictograms have been adapted for South Africans,^6^ while other researchers have developed pictograms specifically for Thai and Sudanese populations.^7^^,^^8^

One of the bases for the use of pictograms lies in the fact that they are supposed to be universally interpretable i.e., they should mean the same thing to all individuals irrespective of culture, language or educational level.^5^ Despite this, many types of pictograms are poorly comprehended by patients and their impact on medication knowledge is inconsistent.^9^ Several studies have shown that many standard pictograms are poorly understandable by individuals in non-Caucasian countries^10^^,^^11^ or by minority populations.^5^ This might be because many of these pictograms were designed within western societies and might not be fully understandable when they are used in other contexts.

Although simply written medicines information in local languages, with pictograms to aid understanding, is desired by patients/consumers in Asia and Africa,^12^ not much is known about the guessability (ability to correctly guess meaning) of standard pharmaceutical pictograms within many of these settings. In Nigeria, the only available study to assess understanding of pictograms in patients, reported than the mean initial guessability score for selected FIP pictograms was 84.5%, which fell below the recommended threshold of 85%.^13^ However, this study only surveyed 40 participants, assessed guessability of only one type of pictogram (FIP) and was conducted in a single hospital in the Southwest. Consequently, the aim of this study was to evaluate the guessability of selected standard pharmaceutical pictograms (FIP & USP) in members of the Nigerian public.

## 2 Methods

### 2.1 Study site

Kaduna metropolis is the capital of Kaduna state, located within the northwestern region of Nigeria. Kaduna is one of the largest states in Nigeria, with a land mass of forty-six thousand and fifty-three (46,053) kilometers square.^14^ It is made up of 23 local government areas and three senatorial districts namely: Kaduna North, Kaduna Central and Kaduna South. The estimated population of the state as of 2020 was 9.48 million.^14^

### 2.2 Study design

The study was cross-sectional and used structured interviews to collect data from conveniently sampled members of the public from May–August 2021.

### 2.3 Study population and sample size

The study was conducted on members of Nigerian public residing in Kaduna metropolis. To be eligible to participate, respondents had to be aged at least 18 years or older, understand and speak English/Pidgin English, have self-declared normal eyesight, not be healthcare professionals or students studying a healthcare related course and be willing to participate. All others were excluded. The sample size for the study was calculated using the Raosoft online sample size calculator which recommended a sample size of 377 (Based on the assumptions that the size of the population to be studied was over 20,000 persons, an acceptable margin of error was 5% with a 95% confidence interval and a response distribution of 50%).

### 2.4 Data collection instruments

Selected pictograms from the United States Pharmacopoeia (USP) and International Pharmaceutical Federation (FIP) pictograms banks were used to collect data for the study.^15^^,^^16^ Selected pictograms were those meant to convey information about drug indications/side-effects, routes of administration, frequency of administration and usage precautions. A total of 24 of these pictograms were selected from the FIP pictogram bank, and 22 from the USP pictogram bank (Appendices I & II). The pictograms were grouped based on the functions mentioned above and printed out on two different A3 sized sheets of paper that were used for the interviews.

A data collection form (Appendix III) was also designed for the study. This form collected information about the respondents' sociodemographic characteristics including gender, age and highest educational level completed etc.

### 2.5 Data collection

Study respondents were approached in a wide variety of public places including offices, hospital waiting rooms, community pharmacies and shops. The aim of the study was explained to them after which they were invited to participate. If they agreed to participate, they were then asked a few questions to ascertain their eligibility. If they were found to be eligible, the researchers would then give them one of the A3 sheets containing either the 24 FIP or 22 USP pictograms, allow them to study it and then ask them to guess the meanings (in their own words) of all the pictograms depicted on the sheet. They were also asked to rate each pictogram as either easy or difficult to understand and make suggestions on how to improve any 3 pictograms they perceived as difficult to understand. All their answers were written down verbatim.

### 2.6 Ethical considerations

Ethical approval was sought for and obtained from Committee on the Use of Human Subjects for Research of Ahmadu Bello University before the commencement of the study (*Approval no*.: ABUCUHSR/2021/UG/005). All the participants were informed about the study objectives and informed that participation was voluntary. Verbal consent was also obtained from each participant before the interviews.

### 2.7 Data analysis

Data obtained from these interviews were coded and entered into the Statistical Package for the Social Sciences (SPSS) version 22 software, which was used for analyses.

Participants' responses were evaluated by two judges who knew the correct meanings of the pictograms. Their task was to decide independently, whether the participants' responses matched the intended meanings of pictograms. They independently assigned scores of “1” to correct responses and a score of “0” to incorrect ones and “I don't know” responses. To ensure the reliability of this process, inter-rater reliability was calculated and reached 98%. In the few cases of disagreement, a third judge who was knowledgeable about the meaning of the pictograms was co-opted, and his decision used to break the tie.

Two different parameters were calculated and used for further analyses: guessability & guessing performance scores. Guessability represented the percentage of correct responses obtained for each individual pictogram, while guessing performance indicated the total number of correct guesses per participant.

Descriptive and inferential statistics (Student *t*-tests and/ One way ANOVA with Tukey's Posthoc tests where required and Pearsons correlation) were used to report the results obtained. Statistical significance level was set at *p* < 0.05.

## 3 Results

### 3.1 Sociodemographic characteristics of study respondents

Data was collected from 400 respondents in total. Two hundred respondents each assessed the selected FIP and USP pictograms, and their socio-demographic characteristics are reported below in Table 1. Respondents that assessed the FIP pictograms were mainly females (57%) and aged between 21 and 30 years of age (38%). Most of them were also educated and had four or more Years Post-Secondary Education as their highest educational qualification (34.5%). Only 20.6% of them reported using long term medication (Table 1). Socio-demographic characteristics of the study respondents that assessed the USP pictograms is also reported in Table 1. Majority of these participants were also female (55.5%) and aged between 21 and 30 years of age (35.5%).

**Table 1:** Socio-demographic characteristics of respondents that assessed the pictograms (*n* = 400).

Characteristic	Variable	FIP	USP
		n (%)	n (%)
Gender	Female	114 (57)	111 (55.5)
	Male	86 (43)	89 (44.5)
Age	18–20 years	23 (11.5)	25 (12.5)
	21–30 years	76 (38)	71 (35.5)
	31–40 years	54 (27)	45 (22.5)
	41–50 years	27 (13.5)	36 (18)
	51–60 years	17 (8.5)	20 (10)
	Above 60 years	3 (1.5)	3 (1.5)
Highest educational level completed	No formal education	3 (1.5)	5 (2.5)⁎
	Primary School	10 (5)	14 (7)
	Junior Secondary School	3 (1.5)	3 (1.5)
	Senior Secondary School	53 (26.5)	57 (28.6)
	2 Years Post-Secondary Education	31 (15.5)	31 (15.6)
	4 or more Years Post-Secondary Education	69 (34.5)	61 (30.7)
	Postgraduate qualification	31 (15.5)	28 (14.1)
Using long-term medication	Yes	41 (20.6)⁎	52 (26)
	No	158 (79.4)	148 (74)

⁎ Values in this row do not sum up to the total because of missing values.

### 3.2 Assessment of the selected FIP pictograms

Number of respondents that correctly guessed the meaning/guessability of the assessed FIP pictograms ranged from 3.5 to 95% (Table 2). The pictogram that majority of respondents were able to correctly guess was ‘Take 1 tablet by mouth’, while the pictogram they were least able to correctly guess was ‘buccale’ (Table 2). Eleven pictograms (45.8%) achieved the International Standards Organization (ISO) comprehensibility cut-off point of 67%, while only 6 pictograms (25%) attained the American National Standards Institute (ANSI) comprehensibility cut-off point of 85% (Table 2).

**Table 2:** Interpretation of the selected FIP pictograms (*n* = 200).

Pictogram no.	Intended meaning	Correct guesses n (%)
P1	Diarrhoea	118 (59)
P2	Vomiting	181 (90.5)⁎⁎
P3	Constipation	26 (13)
P4	Dizziness	58 (29)
P5	Headache	179 (89.5)⁎⁎
P6	Take 1 tablet by mouth	190 (95)⁎⁎
P7	Inhale	125 (62.5)
P8	Suppository	100 (50)
P9	1 drop in right eye	161 (80.5)⁎
P10	Inject subcutaneously	137 (68.5)⁎
P11	Buccale	7 (3.5)
P12	Take this medicine in the morning	136 (68)⁎
P13	Take this medicine at night	181 (90.5)⁎⁎
P14	Take 1 capsule every 6 h	33 (16.5)
P15	If breastfeeding- do not take medication	107 (53.5)
P16	Do not drive	139 (69.5)⁎
P17	Keep medication in the fridge	105 (52.5)
P18	Do not take with alcohol or drug	105 (52.5)
P19	Do not take when pregnant	177 (88.5)⁎⁎
P20	Do not crush	100 (50)
P21	Keep out of reach of babies	64 (32)
P22	Shake this medicine before use	90 (45)
P23	Take with food	135 (67.5)⁎
P24	Take with water	173 (86.5)⁎⁎

⁎ Achieved at least 67% comprehensibility.

⁎⁎ Achieved at least 85% comprehensibility.

The mean guessability score varied across the different pictogram categories (Table 3). The categories with the highest means were routes of drug administration and precautions with mean percentage scores of 60, although the overall mean percentage was 58.9% (Table 3).

**Table 3:** Interpretation of the selected FIP pictograms by category.

Category (number of pictograms)	Mean	Standard deviation	Min – Max
Indications and side effects (5)	56.2	35.0	13–90.5
Routes of drug administration (6)	60	31.7	3.5–95
Frequency of administration (3)	58.3	37.9	16.5–90.5
Precautions (10)	60	18.0	32–88.5
*Total (24)*	*58.9*	*26.2*	*3.5–95*

### 3.3 Assessment of the selected USP pictograms

Number of respondents that correctly guessed the meaning/guessability of the assessed USP pictograms ranged from 27.5 to 97% (Table 4). The pictogram that majority of respondents were able to correctly guess was ‘take by mouth’, while the pictogram they were least able to correctly guess was ‘this medicine may make you dizzy’ (Table 4). Thirteen pictograms (59%) achieved the International Standards Organization (ISO) comprehensibility cut-off point of 67%, while only 6 pictograms (27%) attained the American National Standards Institute (ANSI) comprehensibility cut-off point of 85% (Table 4).

**Table 4:** Interpretation of the Selected USP Pictograms (*n* = 200).

Pictogram no.	Intended meaning	Correct guesses n (%)
P1	For stomach/ intestinal problems	105 (52.5)
P2	This medicine may make you dizzy	55 (27.5)
P3	For Headache	178 (89)⁎⁎
P4	Take by mouth	194 (97)⁎⁎
P5	Inhaler	134 (67)⁎
P6	Insert into rectum	77 (38.5)
P7	Place drops in lower eyelid	187 (93.5)⁎⁎
P8	Injection	178 (89)⁎⁎
P9	Dissolve under the tongue	67 (33.5)
P10	Take in the morning	153 (76.5)⁎
P11	Take at bedtime	162 (81)⁎
P12	Take 4 times a day	91 (45.5)
P13	Do not take if breastfeeding	138 (69)⁎
P14	Store in the refrigerator	154 (77)⁎
P15	Do not drive if this medicine makes you sleepy	108 (54)
P16	Do not drink alcohol while taking this medication	56 (28)
P17	Do not take if pregnant	176 (88)⁎⁎
P18	Do not break or crush tablets or open capsule	79 (39.5)
P19	Do not store medicine where children can reach it	169 (84.5)⁎
P20	Shake well	89 (44.5)
P21	Take with meals	151 (75.5)⁎
P22	Take with water	170 (85)⁎⁎

⁎ Achieved at least 67% comprehensibility.

⁎⁎ Achieved at least 85% comprehensibility.

The mean guessability score for the USP pictograms also varied across the different pictogram categories (Table 5). The categories with the highest means were routes and frequency of drug administration with mean percentage scores of 69.6 and 67.7, although the overall mean percentage was 65.3% (Table 5).

**Table 5:** Interpretation of the selected USP pictograms by category.

Category (number)	Mean	Standard deviation	Min - Max
Indications and side effects (3)	56.3	30.9	27.5–89
Routes of drug administration (6)	69.6	28.2	33.5–97
Frequency of administration (3)	67.7	19.3	45.5–81
Precautions (10)	64.5	21.4	28–88
*Total (22)*	*65.3*	*23.1*	*27.5–97*

### 3.4 Associations between guessing performance scores of study respondents and their socio-demographic characteristics

The total number of FIP pictograms correctly identified by study respondents (Their guessing performance scores) ranged from 5 to 23, with a mean ± Standard deviation (SD) of 14.1 ± 3.7.

When Student T-tests and One-way ANOVA were used to compare mean guessing performance scores across respondents' socio-demographic characteristics, significant differences were seen in the highest educational level completed and age categories (Table 6). A Tukey's post hoc test showed that the significant score variations for the highest educational level category were between respondents who had only completed primary education and those who had completed 2-years post-secondary education (*p* = 0.020), 4 years-post-secondary education (*p* = 0.014) and those with postgraduate degrees (*p* = 0.021). A negative weak-moderate correlation (*r* = −0.24, *p* = 0.001) was also seen between age and guessing performance scores.

**Table 6:** Associations between socio-demographic characteristics of study respondents and their total guessing performance scores for the FIP pictograms.

Characteristic	Variable	Mean score	*p*-value
Gender	Female	14.4	0.308
	Male	13.8	
Age	18–20 years	15.1	0.044⁎
	21–30 years	14.4	
	31–40 years	14.5	
	41–50 years	13.4	
	51–60 years	12.5	
	Above 60 years	9.7	
Highest educational level completed	No formal education	10.3	0.003⁎
	Primary School	10.5	
	Junior Secondary School	10.7	
	Senior Secondary School	13.8	
	2 Years Post-Secondary Education	14.8	
	4 or more Years Post-Secondary Education	14.6	
	Postgraduate qualification	14.8	
⁎Using long-term medication	Yes	14.1	0.837
	No	14.2	

⁎ Significant at *p* < 0.05.

The total number of USP pictograms correctly identified by study respondents (Their guessing performance scores) ranged from 4 to 22, with a mean ± Standard deviation (SD) of 14.4 ± 3.8.

When Student *t*-tests and One-way ANOVA were used to compare mean USP guessing performance scores across respondents' socio-demographic characteristics, the only significant difference was seen in the highest educational level completed category (Table 7). A Tukey's post hoc test showed that the significant score variations were between respondents who had postgraduate degrees and those who had no formal education (*p* = 0.005), those who had only completed primary education (*p* = 0.001), and those who had only completed senior secondary school (*p* = 0.038). A negative weak correlation (*r* = −0.113) was also seen between age and guessing performance scores, but the correlation was not statistically significant.

**Table 7:** Associations between Socio-demographic Characteristics of Study Respondents and their Total Guessing Performance Scores for the USP Pictograms.

Characteristic	Variable	Mean score	*p*-value
Gender	Female	14.6	0.360
	Male	14.1	
Age	18–20 years	14.2	0.398
	21–30 years	14.8	
	31–40 years	14.9	
	41–50 years	13.7	
	51–60 years	13.1	
	Above 60 years	14.3	
Highest educational level completed	No formal education	10.2	<0.001⁎
	Primary School	11.6	
	Junior Secondary School	11	
	Senior Secondary School	14.2	
	2 Years Post-Secondary Education e.g., NCE/OND	14.7	
	4 or more Years Post-Secondary Education e.g., HND/BSc	14.5	
	Postgraduate qualification	16.7	
⁎Using long-term medication	Yes	14.6	0.540
	No	14.3	

⁎ Significant at p < 0.05.

## 4 Discussion

This study evaluated the guessability of standard pharmaceutical pictograms in members of Nigerian public residing in Kaduna state and assessed associations between respondents' socio-demographic characteristics and their guessing performance. Results obtained showed that there were variations in guessability for the pictograms, although the USP pictograms had higher guessability rates on average than the FIP pictograms. Highest educational level completed was also associated with guessing performance on both types of pictograms although age was also associated with guessing performance on the FIP pictograms.

According to the International Organization for Standardization (ISO), pictograms should be understood by at least 67% of participants before they can be thought to be valid.^17^ In this study, less than half of the selected FIP pictograms achieved this comprehensibility cut-off, which is similar to findings by Malhotra et al.^18^ in older Singaporean adults where more than half of the FIP pictograms they tested also did not reach the ISO cutoff. However, for the USP pictograms tested in this study, a little over half (13 out of 22) reached this ISO cutoff point which is like estimates from other studies conducted on members of the public in Portugal and Iran.^11^^,^^19^ Still on standards, the American National Standard Institute (ANSI) recommends that for pictograms to be thought to be comprehensible, they should be understood by 85% of participants.^20^ As would be expected, only six each of the FIP and USP pictograms assessed during this study reached this much higher cutoff point, and a very similar trend has been reported in several other studies.^11^^,^^19^^,^^21^ These findings suggest that while some of these pictograms can be adopted and used as they are, quite a few them will need to be redesigned before they can be optimally used within the Nigerian setting.

Guessability varied across pictogram categories and by pictogram type (FIP or USP) in this study. For the FIP pictograms, the highest mean guessability scores were found in the pictograms depicting routes and frequency of drug administration and precautions. Good guessability of pictograms depicting routes and frequencies of administration are in line with findings from other studies.^18^^,^^22^ For the USP pictograms, frequency and routes of drug administration categories also had the highest mean guessability scores. These findings may be simply explained by considering that these categories represent medicines information that is routinely provided during medication counselling and thus very familiar to many individuals. For example, the FIP & USP pictograms with highest guessability “take 1 tablet by mouth” or “take by mouth” can be said to be far more familiar to the average individual when compared to the pictograms with least guessability that represented “buccale” and “this medication may make you dizzy”.

In this study, mean overall guessability of the USP pictograms was 65.3% while that of the FIP pictograms was 58.9%. While it is difficult to make head-to-head comparisons, especially since two different groups of respondents assessed the selected FIP and USP pictograms in this study, it would seem that the USP pictograms were generally better understood over the FIP ones. This matches with earlier findings by Kanji et al.^5^ and Xu^23^ that USP pictograms had higher comprehensibility when compared to FIP pictograms in Hindu and Chinese populations respectively.

Several demographic characteristics have been reported in the literature as being associated with pictogram comprehension. Some of the more commonly reported characteristics include age and educational level.^11^^,^^18^^,^^19^^,^^21^^,^^24^ There were statistically significant associations between education level and guessing performance scores for both types of pharmaceutical pictograms in this study. As would be expected, the pictograms were generally better interpreted by respondents who had higher education levels, which correlated with earlier findings by Barros et al.,^20^ Saremi et al.^11^ and Odunfa et al..^13^ This is likely because people with higher education levels have greater ability to decode symbols. There was also a statistically significant negative correlation between age and guessing performance scores for the FIP pictograms. Other studies^11^^,^^19^^,^^21^ have equally reported negative associations between age and pictogram comprehension.

This study has provided further insights on the ability of members of the Nigerian public to comprehend standard pharmaceutical pictograms. These data may be used by several stakeholders including drug companies when designing patient medication leaflets or national regulatory bodies when developing policies/guidelines relating to the presentation of medicines information. Some of the strengths of this study include the number and diversity of respondents interviewed and the study design. Study limitations include potential sampling bias (due to the convenience sampling of respondents) and the fact that we only selected some pictograms and did not assess the entire sets. Further research (preferably of a qualitative nature) may need to be carried out to identify issues with the pictograms that had poor guessability rates and provide recommendations on how to improve them.

## 5 Conclusions

Guessability of both pictogram types (FIP and USP) in members of the Nigerian public varied widely, but the guessability of USP pictograms was generally better than that for the FIP pictograms. Guessing performance of respondents for both pictogram types was also affected by their educational level. While some of these pictograms may be used as they are, many of them need to be redesigned before they can be correctly interpreted by members of the Nigerian public.

## Funding

This research did not receive any specific grant from funding agencies in the public, commercial, or not-for-profit sectors.

## Declaration of Competing Interest

None.

## Appendix A Supplementary data

FIP and USP pictograms tested during the study. Image 1
