# Supplementary material for: Guessability of standard pharmaceutical pictograms in members of the Nigerian public
Source: Explor Res Clin Soc Pharm. 2023 Mar 10;9:100240. doi: 10.1016/j.rcsop.2023.100240 (PMC10040885; doi:10.1016/j.rcsop.2023.100240)
Supplement: Supplementary file 1 — FIP and USP pictograms tested during the study. [file mmc1.docx]

**APPENDICES**

**Appendix I: Selected FIP Pictograms**

| ***INDICATIONS/ SIDE EFFECTS*** | | | | | | | | |
| --- | --- | --- | --- | --- | --- | --- | --- | --- |
| 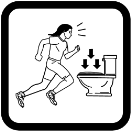1 | 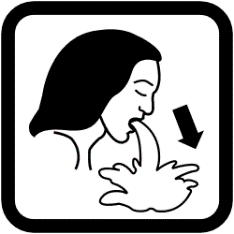  2 | | 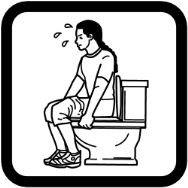 3 | | 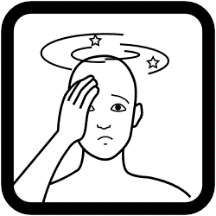 4 | | 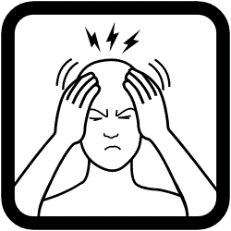  5 | |
| ***ROUTE*** |  | |  | |  | |  | |
| 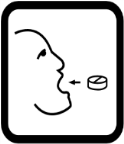 6 | 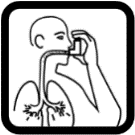7 | | 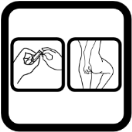8 | | 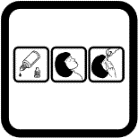  9 | | 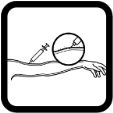  10 | |
| 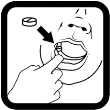11 |  | |  | |  | |  | |
| ***FREQUENCY*** |  | |  | |  | |  | |
| 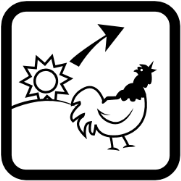12 | 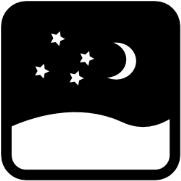13 | | 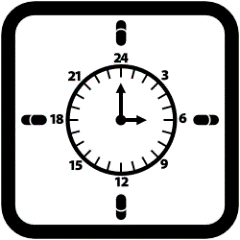  14 | |  | |  | |
| ***PRECAUTIONS*** | | | | | | | | |
| 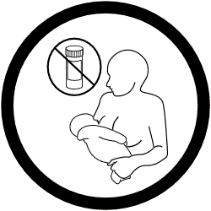  15 | | 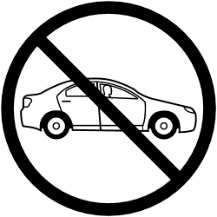  16 | | 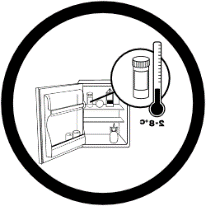  17 | | 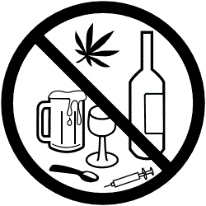  18 | | 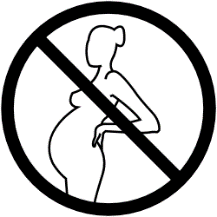  19 |
| 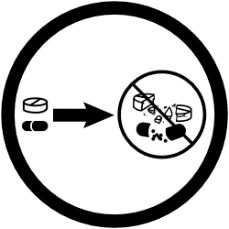  20 | | 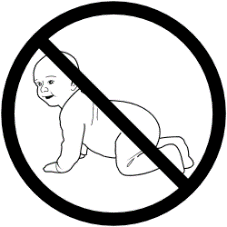  21 | | 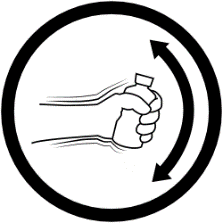  22 | | 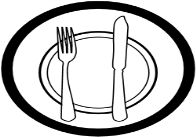23 | | 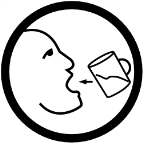24 |

**Appendix II: Selected USP Pictograms**

| INDICATION/ SIDE EFFECTS  1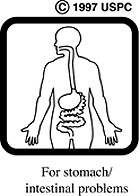 2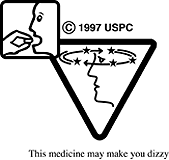3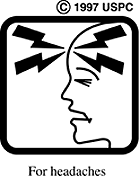 |
| --- |
| ROUTE  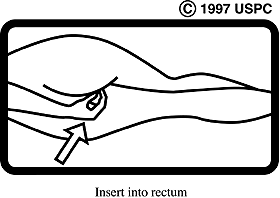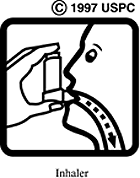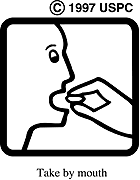 4 5 6      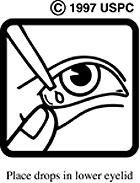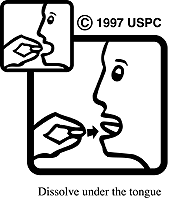  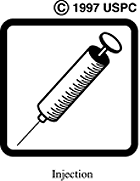  7  8 9 |
| 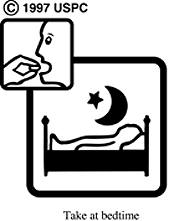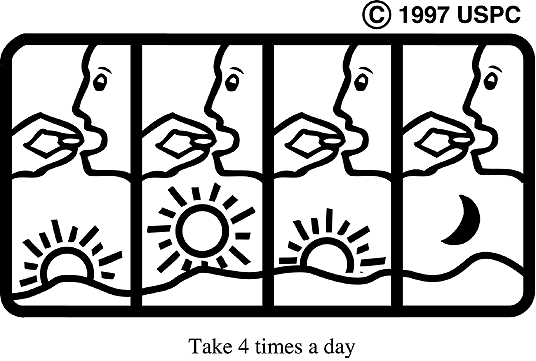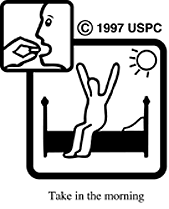 FREQUENCY 11 12  10 |
| 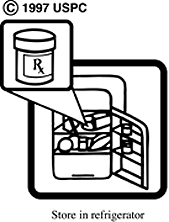PRECAUTIONS 14 15 16  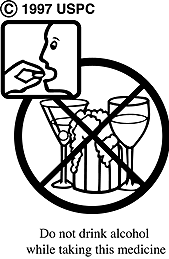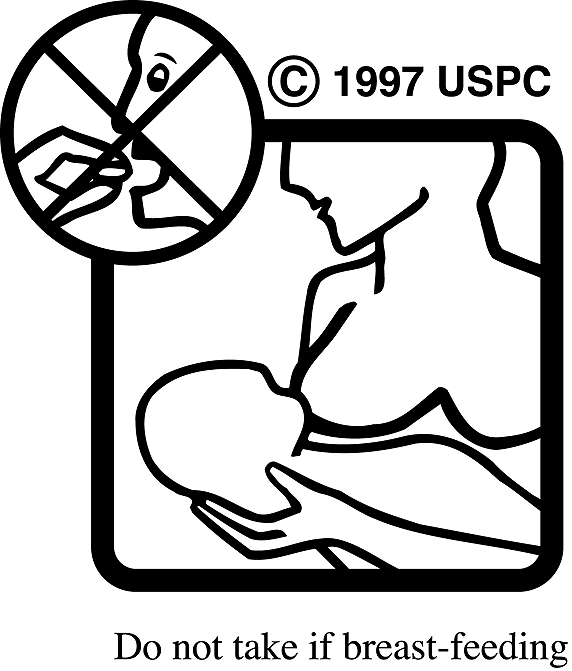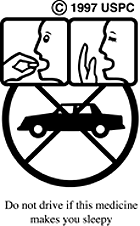 13        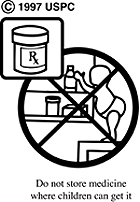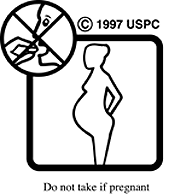  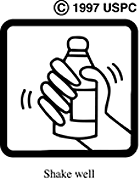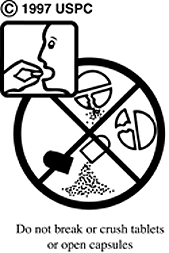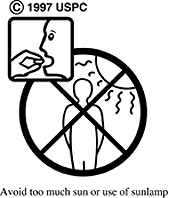      17 18 19 20  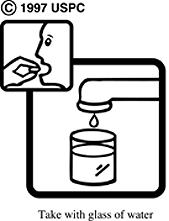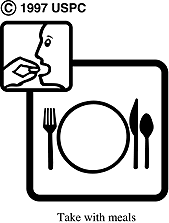  21  22 |

**Appendix III: Data Collection Form**

1. Gender
2. Male
3. Female
4. Age: ………
5. Highest level of education completed:
6. No formal education
7. Primary School
8. Junior Secondary School
9. Senior Secondary School
10. 2 Years Post-Secondary Education
11. 4 or more Years Post-Secondary Education
12. Postgraduate qualification
13. Are you using/on any long-term medication:
14. Yes
15. No
